# Colonic sand impaction with cecal rupture and peritonitis in an adult African savanna elephant, and review of noninfectious causes of gastrointestinal disease in elephants

**DOI:** 10.1177/10406387221130024

**Published:** 2022-11-18

**Authors:** Tamires G. W. Teodoro, Francisco A. Uzal, Nicolas Streitenberger, Monika A. Samol, Eileen E. Henderson, Javier Asin

**Affiliations:** Department of Veterinary Clinics, School of Veterinary Medicine and Animal Science, Paulista State University, Botucatu, Brazil; California Animal Health and Food Safety Laboratory System, San Bernardino Laboratory, School of Veterinary Medicine, University of California–Davis, Davis, CA, USA; California Animal Health and Food Safety Laboratory System, San Bernardino Laboratory, School of Veterinary Medicine, University of California–Davis, Davis, CA, USA; California Animal Health and Food Safety Laboratory System, San Bernardino Laboratory, School of Veterinary Medicine, University of California–Davis, Davis, CA, USA; California Animal Health and Food Safety Laboratory System, San Bernardino Laboratory, School of Veterinary Medicine, University of California–Davis, Davis, CA, USA; California Animal Health and Food Safety Laboratory System, San Bernardino Laboratory, School of Veterinary Medicine, University of California–Davis, Davis, CA, USA

**Keywords:** cecal rupture, colic, elephants, obstruction, sand

## Abstract

Gastrointestinal disorders are among the most common disease processes in captive elephants. Colic is a frequent clinical presentation and may have several infectious and noninfectious causes. Ingestion of sand has been reported in elephants living in enclosures with loose sandy soils. Similar to the situation in horses, sand ingestion can cause intestinal impaction and colic in elephants. Here we describe a case of colonic sand impaction with cecal rupture and peritonitis in an African savanna elephant from a zoologic collection that died after several days of colic. On autopsy, abundant, gritty, sandy material was found within a segment of colon immediately aboral to the cecum. There was a full-thickness tear in the cecal wall, free intestinal contents within the abdominal cavity, and peritonitis. To our knowledge, the postmortem examination of an elephant with sand impaction and cecal rupture has not been reported previously; this condition should be included among the differential diagnoses in elephants with colic. We review the reports of noninfectious causes of gastrointestinal disease in elephants, which include cases of small intestinal and colonic torsion and of intestinal obstruction by fecal boluses.

African elephants are proboscidean mammals comprised of 2 species based on their genetic background: the African savanna (or bush) elephant (*Loxodonta africana*) and the African forest elephant (*Loxodonta cyclotis*), which, until recently, were considered subspecies of the same species.^25,27^ Together with the Asian elephant (*Elephas maximus*), these are the largest animals kept in captivity in zoologic collections.^15^

Elephants are monogastric, herbivorous, non-ruminant, hindgut fermenters with a gastrointestinal (GI) metabolism reminiscent of that of horses. In fact, elephants and horses have a similar alimentary system that includes a simple stomach, small intestine, cecum, colon, and rectum; however, compared to horses and other herbivores, elephants have a proportionally shorter digestive tract, with a less capacious cecum and a larger colon without prominent sacculations.^6,15^ Elephants are mixed feeders and consume grass, leaves, branches, and tree bark.^18^ The African elephant is more adapted to a leaf-rich diet, whereas the Asian elephant prefers a predominantly grass-based diet; some authors have suggested that the latter has a longer alimentary canal.^6,15^ In general, elephants consume 1.5–2% of their body weight in dry matter daily and spend up to 80% of their day feeding.^27^

GI disorders are the most common causes of morbidity and mortality in captive elephants, and signs of colic are reported commonly.^4,21^ Colic is a broad clinical term that refers to abdominal pain with associated clinical signs such as restlessness, stretching, rolling, and abdominal distension. Colic is observed frequently in horses and several other zoologic large species, such as rhinoceroses^16,22^; however, compared to horses, elephants with colic often exhibit subtler signs of discomfort.^15^ In elephants, colic is associated with a variety of infectious agents, including *Salmonella* spp., *Clostridium* spp., elephant endotheliotropic herpesvirus (*Elephantid betaherpesvirus 1*), and helminth parasites^2,3,5,13,19^; or with noninfectious causes, including partial or complete intestinal tract obstruction as a result of intestinal displacements, impaction, or foreign bodies.^10,14,17,24,29,30^ Dental disease can predispose to some of the latter conditions,^12^ and cholelithiasis has also been related to colic-like signs in elephants.^8^

Here we present the results of the postmortem examination of an African savanna elephant that had sand colic with colonic impaction, and secondary cecal rupture with peritonitis. To our knowledge, there are no similar descriptions available in the scientific literature in English. We also review the literature on noninfectious GI disorders leading to colic in elephants with a focus on gross and microscopic pathologic findings. With this report and the associated review, we intend to provide data of value for other diagnostic veterinary pathologists who perform elephant autopsies in the future.

A 39-y-old female African savanna elephant from a regional zoologic collection was submitted for autopsy to the San Bernardino branch of the California Animal Health and Food Safety Laboratory System (CAHFS; University of California–Davis). The animal had a 17-d history of GI disease, ventral edema, inappetence, mild hyperthermia, and colic including holding one foot off the ground, lying down and getting up, and refusing food and water. Hematologic and biochemical abnormalities included heterophilia with a left shift, elevated activities of lactate dehydrogenase and creatine kinase, and increased serum amyloid A concentration. Enrofloxacin, flunixin meglumine, and omeprazole were administered; however, the elephant was subsequently found dead in its night stall. The owner reported a history of recurrent bouts of colic in the past, prior to the 17-d episode that led to its death, and that included passing large amounts of gravel in the stool.

Grossly, the carcass was in good nutritional condition, well-fleshed with ample fat reserves, mildly dehydrated, and in a mild state of postmortem decomposition. There were numerous, 2–4-cm diameter skin abrasions and ulcers on the ventral abdomen. Moderate, focally extensive, subcutaneous edema was observed in the caudal region of the ventral abdomen and inguinal area.

The abdominal cavity contained ~10 L of turbid, yellow-to-light-red fluid with strands of fibrin and free vegetal fragments. The abdominal serosae were diffusely dark-red, with petechiae and loosely attached aggregates of fibrin and fragments of vegetal digesta (Fig. 1A). There was a single, 25-cm transmural tear in the apical third of the cecal wall, with raised, thickened, firm, dark-red edges, and regionally extensive hemorrhage with fibrin and digesta attached to the adjacent cecal serosa (Fig. 1B). Cecal contents consisted of long semi-dry fragments of fibrous plant material. The surrounding cecal mucosa was diffusely gray and mildly edematous. An ~75-cm segment of the colon immediately aboral to the cecum contained abundant, gritty, very compacted, sandy material with small irregular stones mixed with plant material, grain, and a moderate amount of brown fluid (Fig. 1C). The content of the remaining colon consisted of densely packed vegetal fibrous material, scant grain, and minimal fluid; the mucosa was diffusely gray. Feces in the rectum were well-formed. Additionally, the lungs were diffusely dark-red and spongy, there were petechiae on the epicardium, and coalescing, 0.5–1-cm, cauliflower-like, exophytic growths on the serosa of both uterine horns.

**Figure 1.**
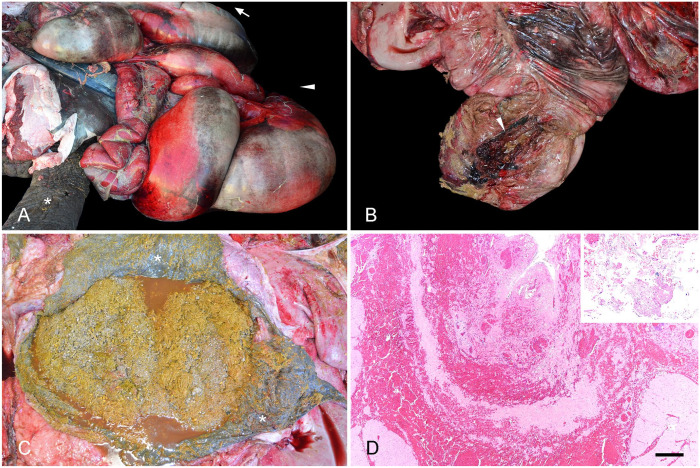
Gross and microscopic findings in an adult African savanna elephant (*Loxodonta africana*) with colonic sand impaction, cecal rupture, and peritonitis. **A.** Ventrolateral view of the carcass with exposed viscera. Peritonitis with diffusely reddened intestinal serosae, hemorrhages, and aggregates of fibrin and plant material. Carcass position: the carcass is lying on its left side, head to the right (asterisk on left hindlimb; arrowhead pointing to ventral side; arrow pointing to the head). **B.** Large tear (arrowhead) in the distal aspect of the cecum, with raised, dark-red borders. The adjacent serosa is reddened and overlaid by fibrin and plant material. **C.** Colon impacted with abundant sandy material mixed with fibrous colonic content and scant fluid. Edematous colonic mucosa (asterisks). **D.** Border of the cecal tear, with hemorrhage and fibrin dissecting the cecal muscularis and expanding the serosa. Bar = 200 μm. Inset: fibrin and vegetal material in the cecal serosa. H&E.

A diagnostic workup following CAHFS standard operating procedures was performed and included: bacterial aerobic culture of the liver, lung, spleen, mesenteric lymph node, peritoneum, cecal wall around the edges of the tear, and all intestinal segments (i.e., small intestine, cecum, colon); anaerobic bacterial culture of all intestinal segments; *Salmonella* spp. enrichment culture of the liver, mesenteric lymph node, peritoneum, and all intestinal segments; *Clostridioides difficile* enrichment culture of all intestinal segments; *Clostridium perfringens* and *C. difficile* toxin ELISAs on content from all intestinal segments; fecal flotation parasitologic examination; and heavy metal screen (including arsenic, cadmium, copper, iron, lead, manganese, mercury, molybdenum, zinc) and selenium concentration determination on the liver. Samples from lung, liver, lymph nodes, mesenteric fat, adrenal gland, kidney, trachea, heart, spleen, haired skin, aorta, skeletal muscle, urinary bladder, mammary gland, tongue, stomach, small intestine, colon, cecum, uterus, and brain were fixed by immersion in 10% neutral-buffered formalin and processed routinely to produce 4-μm thick, H&E-stained sections.

Histologically, the borders of the cecal tear had hemorrhage, necrosis, fibrin, heterophil-rich inflammatory infiltrates with fewer lymphocytes, plasma cells, macrophages, and fibrinoid necrosis of vessel walls with occasional thrombi (Fig. 1D). Areas of cecum distant to the borders of the tear had hemorrhages dissecting the muscle layers, and submucosal edema. The serosae of various GI organs, the mesentery, and the hepatic and splenic capsules were overlaid by fibrin, heterophils, debris, bacterial colonies, and plant material. In addition, there was pulmonary congestion with rare thrombosis, and hepatic centrilobular congestion, most likely related to sepsis and/or endotoxemia. Mild pneumoconiosis, hepatic and splenic hemosiderosis, and mild renal glomerular capsule fibrosis with rare mineralization were also detected and considered incidental and/or age-related findings. Histologically, the uterine growths were consistent with serosal fibropapillomas, which are frequent incidental findings in this location in aged elephants.^20^

Mixed growth of *Streptococcus lutetiensis*, *Streptococcus equinus*, *Streptococcus* sp., *Pediococcus pentosaceus*, and *Lactobacillus* spp. was obtained in the aerobic cultures of the liver, mesenteric lymph node, intestines, and cecal wall around the edges of the tear. *Enterococcus faecium* in mixed culture was isolated from the peritoneum. *Clostridium bifermentans* and *C. perfringens* were obtained in the anaerobic cultures of the small intestine and cecum, respectively, combined with mixed flora. *C. perfringens* and *C. difficile* toxin ELISAs, and *C. difficile* and *Salmonella* spp. enrichment cultures, were negative. Fecal flotation did not detect any parasites, parasite eggs, or oocysts. The heavy metal and selenium concentrations were unremarkable when extrapolating to results in other domestic species.

Sand impaction in the colon in our case most likely caused partial obstruction, leading to secondary cecal rupture as a result of reduced outflow and increased intraluminal pressure. The changes detected histologically in the vessels along the cecal tear would suggest that vascular compromise (i.e., compression of veins following distention, interference with venous return, marked congestion, ischemia) was also likely involved in the tissue damage. In turn, the cecal rupture allowed leakage of intestinal content into the peritoneal cavity, with subsequent peritonitis and probable endotoxic shock leading to death.

The aerobic bacteria isolated from the liver, mesenteric lymph node, intestines, and cecal tear were considered contaminants, normal GI microbiota, or a combination of both. *E. faecium* is an enterobacterium found commonly in the intestinal tract of various animals.^9^ In our case, *E. faecium* could have been released to the peritoneum through the cecal tear, contributing to peritonitis. *C. bifermentans* is a commensal enteric organism in various species and was thus considered incidental.^23^ *C. perfringens* was isolated from the cecum; however, this was considered to be a nonsignificant finding given that the *C. perfringens* toxin ELISA was negative, and this bacterium can be found in the feces of healthy elephants.^26^

The clinical signs of colic in elephants resemble those seen in horses, including restlessness, stretching, rolling or assuming bowing posture, abdominal distension, lack of defecation, biting the tip of the trunk, groaning, and tenesmus. Affected animals can have intestinal stasis, severe dehydration, electrolyte imbalance, endotoxic shock, and circulatory collapse, which may result in recumbency and death.^10,11^ Similar clinical signs were observed in our case prior to death. Additionally, this elephant had a long history of recurrent bouts of colic with the presence of gravel in the stool, which was most likely associated with sand accumulation in the GI tract prior to the episode that led to its death.

Sand impaction of the colon of horses is common in geographic areas with loose sandy soils; accidental ingestion of sand has been described when horses are fed hay directly on the ground.^7^ Information on sand colic of elephants in the literature is scarce. A 2-y-old Asian elephant from a zoologic collection was treated for GI impaction associated with the ingestion of sand and clay from an area in the enclosure that had been top dressed with clay^29^; it was speculated that sodium deficiency could have led to pica. Plasma sodium concentration was not available for our case, but pica caused by sodium or other deficiencies cannot be ruled out.

Enclosures for elephants must have soil or sand available to assist with thermoregulation, promote normal wear of footpads, and allow for dust bathing, according to the Association of Zoos and Aquariums Standards for Elephant Management and Care 2022.^1^ Furthermore, sand can be used as an environmental enrichment item to increase animal activity and foster exploration of the area^11^; however, the accidental ingestion of large amounts of sand can lead to accumulation of sand in the intestinal tract and impaction. Impaction can cause total or partial obstruction and subsequent increase in the intraluminal pressure in the cranial intestinal segments, with possible rupture, as occurred in our case.

Cecal rupture in horses often occurs as a complication of impaction; 2 distinct types are described: type 1 results from excessive accumulation of dry ingesta, commonly in the large colon; type 2 is believed to be caused by intestinal dysmotility leading to secondary accumulation of fluid and ingesta.^28^ Furthermore, impaction of the cecum or colon in horses causes abnormal motility and may result in altered colonic or cecal peristalsis. In addition, sand impaction in horses is often associated with concurrent displacements, such as torsion.^7,16^ A similar pathophysiology likely occurs in elephants with cecal rupture and impaction, given the anatomic similarities with the equine gut.^6^ As such, we considered the cecal rupture in our case to be type 1.^28^

Only a few cases of GI impaction, strangulation, and other displacements in elephants are described in the literature, which is limited to single case reports or small case series (Table 1). Only 1 of the 8 cases included in our review was an African elephant; the rest were Asian elephants. Ages of the reviewed cases were 5-d to 58-y. Clinical signs in most cases were of colic, with anorexia and abdominal discomfort recorded most commonly. In 4 of 8 cases, the disease process resulted in death or euthanasia of the animal; 3 elephants had either intestinal torsion or strangulating incarcerations that compromised the viability of the involved segments; 1 case had severe esophageal obstruction. Four animals recovered after treatment, which included 3 elephants that were able to pass the obstructing material (i.e., clay or fecal bolus/es) in the feces, as well as one with colonic strangulation caused by congenital fibrous adhesions, which was corrected surgically.

**Table 1.** Reported cases of noninfectious causes of gastrointestinal disease in elephants.

Species	Age	Sex	Clinical history	Outcome	Gross findings	Histologic findings	Ref.
Asian elephant	5 d	M	Hematochezia and respiratory abnormalities following an episode of near drowning	Death	360° clockwise torsion of small intestine, cecum, and large colon	Multifocal hemorrhage in small intestine, and mucosal necrosis	^30^
Asian elephant	8 d	F	Decreased nursing activity, restlessness, tachycardia, discomfort, distended intestinal loops on ultrasound	Survived; surgery for correction and biopsy	Adhesions in distal small intestine with strangulation of large colon	Congenital adhesions; hemorrhage and fibrin in strangulated segment	^30^
Asian elephant	18 mo	F	Clay ingestion and subsequent abdominal pain, inappetence, unusual posture, and tympanic ping on auscultation; low plasma sodium concentration	Survived after treatment, passing clay in feces, and sodium supplementation	NA	NA	^29^
Asian elephant	16 y	F	Distended abdomen, lack of defecation, and no food or water intake further to intestinal impaction and obstruction	Survived after treatment and passing several fecal boluses	NA	NA	^17^
Asian elephant	4 y	CM	Anorexia and diarrhea, abdominal discomfort, tachycardia, tenesmus	Death	Mesenteric rent incarcerating 1.5 m of distal small intestine with ruptured wall and septic peritonitis	Transmural congestion, edema, and mucosal necrosis	^30^
African elephant	22 y	F	Anorexia and mucoid diarrhea after grazing in lush pasture, abdominal distension, discomfort, and recumbency	Death	360° torsion around the root of the mesentery, involving the entire large colon, which was congested	Submucosal edema, hemorrhages, and fibrin thrombi	^30^
Asian elephant	42 y	F	Hyporexia, drooling, dysphagia, mat of plant fibers in the esophagus seen by endoscopy	Death; euthanasia due to poor prognosis	Esophageal obstruction by compacted plant material; mucosal ulceration; plant material, exudate, gas, and hemorrhage dissecting the wall	Early neovascularization, fibrosis, suppuration, mixed bacteria, plant material	^24^
Asian elephant	58 y	F	Constipation, anorexia, and abdominal distension further to complete gastrointestinal obstruction and ileus	Survived after treatment and passing a large fecal bolus	NA	NA	^14^

CM = castrated male; F = female; M = male; NA = not available; U = unknown.

When available, reported gross lesions included evidence of torsion, strangulation, or impaction; histology of the involved segments invariably revealed severe damage to the wall, with congestion, hemorrhage, and necrosis. The causes and predisposing factors in these cases were in general undetermined. Authors speculated that these processes can result from ingestion of sand, clay, dirt, or excessive fiber in a short period, which become impacted in the GI tract, leading to intestinal obstruction and possibly torsion.^10,30^ Causes of intestinal displacements in elephants may include aerophagy and sudden dietary changes, although the role of these factors remains speculative in most cases.^30^
